# Pharmacokinetics of First-Line Drugs in Children With Tuberculosis, Using World Health Organization–Recommended Weight Band Doses and Formulations

**DOI:** 10.1093/cid/ciab725

**Published:** 2021-08-22

**Authors:** Chishala Chabala, Anna Turkova, Anneke C Hesseling, Kevin M Zimba, Marieke van der Zalm, Monica Kapasa, Megan Palmer, Maxwell Chirehwa, Lubbe Wiesner, Eric Wobudeya, Aarti Kinikar, Vidya Mave, Syed Hissar, Louise Choo, Kristen LeBeau, Veronica Mulenga, Robb Aarnoutse, Diana Gibb, Helen McIlleron

**Affiliations:** 1 University of Zambia, School of Medicine, Department of Paediatrics, Lusaka, Zambia; 2 University of Cape Town, Faculty of Health Sciences, Department of Medicine, Division of Clinical Pharmacology, Cape Town, South Africa; 3 University Teaching Hospitals–Children’s Hospital, Lusaka, Zambia; 4 Medical Research Council–Clinical Trials Unit at University College London, Institute of Clinical Trials and Methodology, London, United Kingdom; 5 University of Stellenbosch, Desmond Tutu Tuberculosis Centre, Cape Town, South Africa; 6 Makerere University-Johns Hopkins University Care Ltd, Kampala, Uganda; 7 Byramjee Jeejeebhoy Government Medical College, Pune, India; 8 India Council of Medical Research, National Institute for Research in Tuberculosis, Chennai, India; 9 Radboud University Medical Center, Nijmegen, The Netherlands; 10 Wellcome Centre for Infectious Diseases Research in Africa (CIDRI-Africa), Institute of Infectious Disease and Molecular Medicine, University of Cape Town, Cape Town, South Africa

**Keywords:** antituberculosis drugs, children, dosing, pharmacokinetics, tuberculosis

## Abstract

**Background:**

Dispersible pediatric fixed-dose combination (FDC) tablets delivering higher doses of first-line antituberculosis drugs in World Health Organization–recommended weight bands were introduced in 2015. We report the first pharmacokinetic data for these FDC tablets in Zambian and South African children in the treatment-shortening SHINE trial.

**Methods:**

Children weighing 4.0–7.9, 8.0–11.9, 12.0–15.9, or 16.0–24.9 kg received 1, 2, 3, or 4 tablets daily, respectively (rifampicin/isoniazid/pyrazinamide [75/50/150 mg], with or without 100 mg ethambutol, or rifampicin/isoniazid [75/50 mg]). Children 25.0–36.9 kg received doses recommended for adults <37 kg (300, 150, 800, and 550 mg/d, respectively, for rifampicin, isoniazid, pyrazinamide, and ethambutol). Pharmacokinetics were evaluated after at least 2 weeks of treatment.

**Results:**

In the 77 children evaluated, the median age (interquartile range) was 3.7 (1.4–6.6) years; 40 (52%) were male and 20 (26%) were human immunodeficiency virus positive. The median area under the concentration-time curve from 0 to 24 hours for rifampicin, isoniazid, pyrazinamide, and ethambutol was 32.5 (interquartile range, 20.1–45.1), 16.7 (9.2–25.9), 317 (263–399), and 9.5 (7.5–11.5) mg⋅h/L, respectively, and lower in children than in adults for rifampicin in the 4.0–7.9-, 8–11.9-, and ≥25-kg weight bands, isoniazid in the 4.0–7.9-kg and ≥25-kg weight bands, and ethambutol in all 5 weight bands. Pyrazinamide exposures were similar to those in adults.

**Conclusions:**

Recommended weight band–based FDC doses result in lower drug exposures in children in lower weight bands and in those ≥25 kg (receiving adult doses). Further adjustments to current doses are needed to match current target exposures in adults. The use of ethambutol at the current World Health Organization–recommended doses requires further evaluation.

## Teaser

We evaluated pharmacokinetics of antituberculosis drugs administered as fixed-dose combinations tablets using WHO weight band (WB) dosing tables. Exposures were low for rifampicin and isoniazid in WBs 4.0 <8kg and ≥25kg; 8kg-12kg for rifampicin; and across all WBs for ethambutol.

Tuberculosis treatment regimens in most low- and-middle income countries is standard, based on World Health Organization (WHO) recommendations and delivered by national programs in the public sector. Ensuring optimal treatment is integral to the global strategy to end childhood tuberculosis [1].

Historically, pediatric doses of the first-line antituberculosis drugs were extrapolated from adult doses, using the same milligram per kilogram of body weight doses. Informed by pharmacokinetic studies demonstrating that this approach does not achieve comparable drug exposures in children [2–5], the WHO revised these recommendations for children weighing <25 kg in 2010, increasing the daily doses of isoniazid (H) by 100% to 10 (range, 7–15) mg/kg, rifampicin (R) by 50% to 15 (10–20) mg/kg, and pyrazinamide (Z) to 35 (30–40) mg/kg. It was envisaged that, using the revised doses, exposures in children would approximate those in adults. Ethambutol (E) doses were unchanged, at 20 (range 15–25) mg/kg/d [6, 7], a dose thought to carry minimal risks of ocular toxicity for children [8, 9].

Implementation of the revised first line drug doses was initially challenging because the fixed-dose combination (FDC) tablet available at the time did not deliver the revised drug ratios [10]. New child-friendly dispersible FDC tablets of RHZ (75/50/150 mg) and RH (75/50 mg) became available in 2015 and, following prequalification by WHO, have been rolled out globally. These new FDCs are water dispersible, scored, palatable, and easy to administer [11].

Serum concentrations of antituberculosis drugs predict tuberculosis treatment response and have been reported as surrogate markers for predicting therapeutic success [12, 13]. Hollow-fiber models and dose fractionation studies show the area under the concentration-time curve (AUC) to be associated with efficacy of antituberculosis drugs [14, 15]. AUC and serum peak concentration (C_max_) are closely correlated, and emerging results from pharmacokinetic studies evaluating the revised dosing in children report C_max_ values below the adult reference values [16] and AUC values lower than those reported in adults, particularly for rifampicin and ethambutol [17–19].

Pharmacokinetic measures of antituberculosis drug exposure vary considerably between study populations. Body size, nutritional status, human immunodeficiency virus (HIV) infection, and developing enzyme maturation functions are sources of pharmacokinetic variability in children [20, 21]. *NAT2* acetylator genotype is a key determinant of isoniazid concentrations, and *SLCO1B1* polymorphisms have been associated with rifampicin exposures [4, 5, 22–25]. Other important factors include the type of formulation, dose preparation and administration, drug-drug interactions, and laboratory assay methods used [20, 21].

The revised WHO weight band dosing, using dispersible child-friendly FDCs, simplifies tuberculosis treatment and programmatic implementation, but supporting pharmacokinetic evidence in children is lacking. We describe the pharmacokinetics at steady state in children dosed with this approach in the SHINE trial, and we sought to identify predictors of exposures of first-line antituberculosis drugs.

## METHODS

### Study Population and Design

This pharmacokinetic study was nested in the phase III treatment shortening SHINE trial (SRCTN63579542), a randomized-controlled trial comparing a 4-month versus standard 6-month antituberculosis drug regimens, using revised WHO pediatric weight band dosing and new FDCs in children with or without HIV. SHINE recruited children aged 0–16 years with non-severe tuberculosis in Zambia, South Africa, Uganda, and India. Non-severe tuberculosis was defined as smear-negative tuberculosis, including pulmonary disease confined to one lobe without cavities, intrathoracic lymph node tuberculosis without significant airway obstruction, and extrathoracic tuberculosis lymphadenitis. Screening, recruitment, clinical care, and follow-up procedures are described elsewhere [26]. A subset of children enrolled in the trial were selected consecutively to participate in pharmacokinetic substudies. Here we report on African children enrolled in Zambia and in South Africa.

### Drugs and Dosages

Antituberculosis drugs were administered in weight bands according to the WHO 2015 dosing recommendations [11]. Dispersible pediatric or adult FDC tablets (Macleods Pharmaceuticals) were used. For the 2-month intensive phase, RHZ (75/50/150-mg dispersible tablets), with or without ethambutol (100-mg tablets), was administered in 4 weight bands to children weighing <25 kg. Children ≥25 kg received weight band–based adult doses, using RHZE (150/75/400/275-mg tablets) [27]. In the continuation phase, RH 75/50-mg tablets were used for children <24.9 kg, and RH 150/75-mg tablets for those ≥25 kg (Table 1). Ethambutol use was guided by local tuberculosis treatment recommendations at the time. In South Africa it was indicated if the child was HIV positive or >8 years of age [28]. In Zambia all children received ethambutol regardless of age, disease severity, or HIV status [29]. Daily drug administration was supervised by the caregiver or parent and drug intake documented on treatment cards provided by the trial. Children living with HIV initiated antiretroviral therapy in accordance with national guidelines.

**Table 1.** Daily Doses of Antituberculosis Tablets for Children in the SHINE Trial, Based on World Health Organization Recommendations

Formulations by Weight Band	Intensive Phase		Continuation Phase
Pediatric dispersible formulations for children weighing 4–24.5 kg	HRZ (50/75/150-mg FDC)	E (100 mg)	HR (50/75-mg FDC)
4.0–7.9 kg	1	1	1
8.0–11.9 kg	2	2	2
12.0–15.9 kg	3	3	3
16.0–24.9 kg	4	4	4
Adult formulation and doses used for children weighing ≥25.0 kg	HRZE (75/150/400/275-mg FDC)		HR (75/150-mg FDC)
25–36.9 kg	2		2

Abbreviations: E, ethambutol; FDC, fixed-dose combination tablet; H, isoniazid; R, rifampicin; Z, pyrazinamide.

### Pharmacokinetic Sampling and Laboratory Analysis

Intensive pharmacokinetic sampling was scheduled after at least 2 weeks of antituberculosis treatment. Caregivers were reminded by phone to administer the antituberculosis drugs in the morning (if evening dosing was preferred by caregiver) for at least a week before the pharmacokinetic sampling visit. On the sampling day, drug intake was observed by research staff after an overnight fast, and breakfast was provided at least 2 hours after drug intake, unless the child was distressed, in which case a snack was permitted. The FDC tablets were dispersed in water or administered whole, in keeping with the practice at home, while children receiving the adult formulation swallowed tablets whole.

Serial venous blood samples were obtained before dosing and at 1, 2, 4, 6, 8, and 12 hours after drug intake. Samples were immediately placed on ice before centrifugation within 30 minutes of collection. Separated plasma samples were stored at −80°C until transportation on dry ice for analysis at the Pharmacology laboratory, University of Cape Town, South Africa. Drug concentrations were determined using liquid chromatography–tandem mass spectrometry assays validated over concentration ranges of 0.117–30.0 mg/L for rifampicin, 0.105–25.0 mg/L, for isoniazid, 0.200–80.0 mg/L for pyrazinamide, and 0.0844–5.46 mg/L for ethambutol [18, 19] according to Food Drug Administration and European Medicines Agency guidelines. The accuracy represented as percentage of the nominal concentration (%Nom) of the lower limit of quantification, low, medium, and high-quality controls were 101%–107%, 92%– 05%, 101%–104%, and 97%–107%, respectively, for rifampicin, isoniazid, pyrazinamide, and ethambutol. The precision is represented as percentage of the coefficient of variation (%CV) was below 11% for all analytes during interday and intraday validation. External quality control samples were provided by the University of Nijmegen, the Netherlands.

### Pharmacokinetic and Statistical Analysis

Drug concentrations below the lower limit of quantification were imputed by halving the lower limit of quantification for the respective drug. Stata software (version 16.1; StataCorp) was used to compute the noncompartmental pharmacokinetic measures (including C_max_, half-life, time to C_max_, and elimination rate constant), for statistical tests, and for regression analyses. The 24-hour concentration for each participant was imputed using a regression equation obtained by regressing log-transformed concentration measurements in the terminal phase of the pharmacokinetic curve against the time of sample. The AUC from 0 to 24 hours (AUC_24_) was derived using the linear-log trapezoid rule and summarized by weight band for each drug. For rifampicin, the reference AUC_24_ was equal to or greater than the mean AUC_24_ (38.7 mg⋅h/L) derived from a meta-analysis of adult studies by Stott et al [22]. The AUC_24_ median ranges reported for studies included in a systematic review by Daskapan et al [23] were used for isoniazid (11.6–26.3 mg⋅h/L, excluding 1 study with outlying results [30]), pyrazinamide (233–429 mg⋅h/L), and ethambutol (16–28 mg⋅h/L). Normal values, as described by Alsultan and Peloquin [16] were used for the C_max_ reference ranges: 8–24 mg/L, 3–6 mg/L, 20–60 mg, and 2–6 mg/L, respectively, for rifampicin, isoniazid, pyrazinamide, and ethambutol.

Quantile regression was used to evaluate covariate effects on AUC_24_, after adjusting for the effect of weight band. HIV status, sex, study site, weight-for-age *z* score (WAZ), and weight-for-height *z* score, were each tested for their effect on the AUC_24_, for each drug, in bivariable models including weight band. All covariates with a *P* value <.2 in the bivariate models were retained in the final model. Drug doses (in milligrams per kilogram weight), age, mid–upper arm circumference, and the mode of drug administration (dispersed in water, swallowed whole, or other) were not included in these models, because they were strongly correlated with weight band.

### Ethical and Regulatory Approvals

The SHINE trial including the pharmacokinetic substudy received regulatory and ethical approvals in Zambia and South Africa. Signed informed consent was obtained from parents or caregivers for this pharmacokinetic substudy.

## RESULTS

Seventy-seven children (43 Zambian and 34 South African) underwent intensive pharmacokinetic sampling. Their median age (interquartile range [IQR]) was 3.7 (1.4–6.6) years, 40 (52%) were male, and 20 (26%) were living with HIV, with 18 receiving antiretroviral therapy (15 receiving efavirenz- and 3 lopinavir-ritonavir–based regimens) at the time of sampling (after a median [IQR] 7 [6–19] weeks of antituberculosis treatment). Patient and treatment characteristics are summarized in Table 2. An overnight fast was reported for all but 2 infants before the intensive pharmacokinetic sampling. Most children received dispersible pediatric FDCs (n = 63, 82%); 14 (18%) of the children ≥25 kg received adult FDCs).

**Table 2.** Participant Characteristics at Time of Pharmacokinetic Sampling, by Weight Band in Children Treated for Tuberculosis

	Weight Band, kg					
Characteristic	4.0–7.9 (n = 16)	8.0–11.9 (n = 14)	12.0–15.9 (n = 16)	16.0–24.9 (n = 16)	≥25–36.9 (n = 15)	All (N = 77)
Age, median (IQR), y	0.6 (0.4–0.8)	1.4 (1.2–2.2)	3.7 (2.4–4.6)	5.8 (5.5–6.7)	11.3 (10.4–12.1)	3.7 (1.4–6.6)
Male sex, no.	8	7	9	5	11	40 (52%)
HIV positive, no.	4	2	5	3	6	20 (26%)
Anthropometric measurements, median (IQR)						
Weight, kg	7.1 (6.8–7.7)	9.1 (8.7–10.2)	14.0 (12.8–14.6)	18.7 (16.6–20.6)	28.5 (28.3–33.7)	14.0 (8.7–20.6)
WAZ	−1.6 (−2.3 to 0.0)	−2.2 (−3.0 to −1.6)	−1.3 (−2.5 to −0.4)	−1.0 (−2.1to 0.2)	−1.4 (−2.1 to −0.6)	−1.5 (−2.3 to −0.4)
WHZ	0.4 (−0.9 to 1.3)	−0.8 (−1.8 to −0.4)	−0.6 (−0.7 to 1.4)	0.2 (−1.2 to 0.6)	…	−0.2 (−1.2 to 0.8)
MUAC, cm	13.9 (12.5–14.3)	14.3 (13.0–14.8)	15.5 (14.7–16.5)	16.9 (15.7–18.1)	18.5 (17.8–20.4)	15.3 (14.0–17.8)
Duration of tuberculosis treatment, median (IQR), wk	6 (5–8)	6 (5–7)	14 (7–20)	16 (6–23)	14 (5–14)	7 (6–19)
Mode of drug administration, no.						
Dispersed	14	11	7	7	1	40 (52%)
Taken whole by mouth	1	2	8	9	14	34 (44%)
Other^a^	1	1	1	0	0	3 (4%)
Dosage of antituberculosis drug, median (IQR), mg/kg						
Rifampicin	10.7 (9.8–12.2)	16.4 (15.4–16.7)	16.1 (15.4–17.2)	15.8 (14.5–17.8)	10.3 (8.8–10.7)	14.6 (10.6–16.9)
Isoniazid	7.1 (6.5–8.1)	10.9 (9.8–11.5)	10.7 (10.3–11.7)	10.6 (9.6–11.8)	5.1 (4.4–5.3)	9.7 (6.5–11.1)
Pyrazinamide	21.4 (19.5–23.4)	32.3 (26.8–33.7)	31.7 (31.3–32.4)	35.1 (26.2–36.1)	28.2 (23.4–28.3)	28.2 (22.7–32.3)
Ethambutol	14.5 (14.1–15.6)	18.7 (17.0–21.7)	21.0 (20.0–22.3)	23.4 (17.1–25.0)	17.8 (16.1–19.4)	18.5 (15.2–21.7)

Abbreviations: HIV, human immunodeficiency virus; IQR, interquartile range; MUAC, mid–upper-arm circumference; WAZ, weight-for-age *z* score; WHZ, weight-for-height *z* score.

^a^Two of 3 children received drugs by nasogastric tube; for 1 child, the mode of administration was not specified

All 77 children had samples analyzed for rifampicin and isoniazid, while 45 children sampled during the intensive phase of treatment contributed pyrazinamide concentrations. Ethambutol was measured in 22 children (all from Zambia) who received it as part of their regimen.

**Figure 1.**
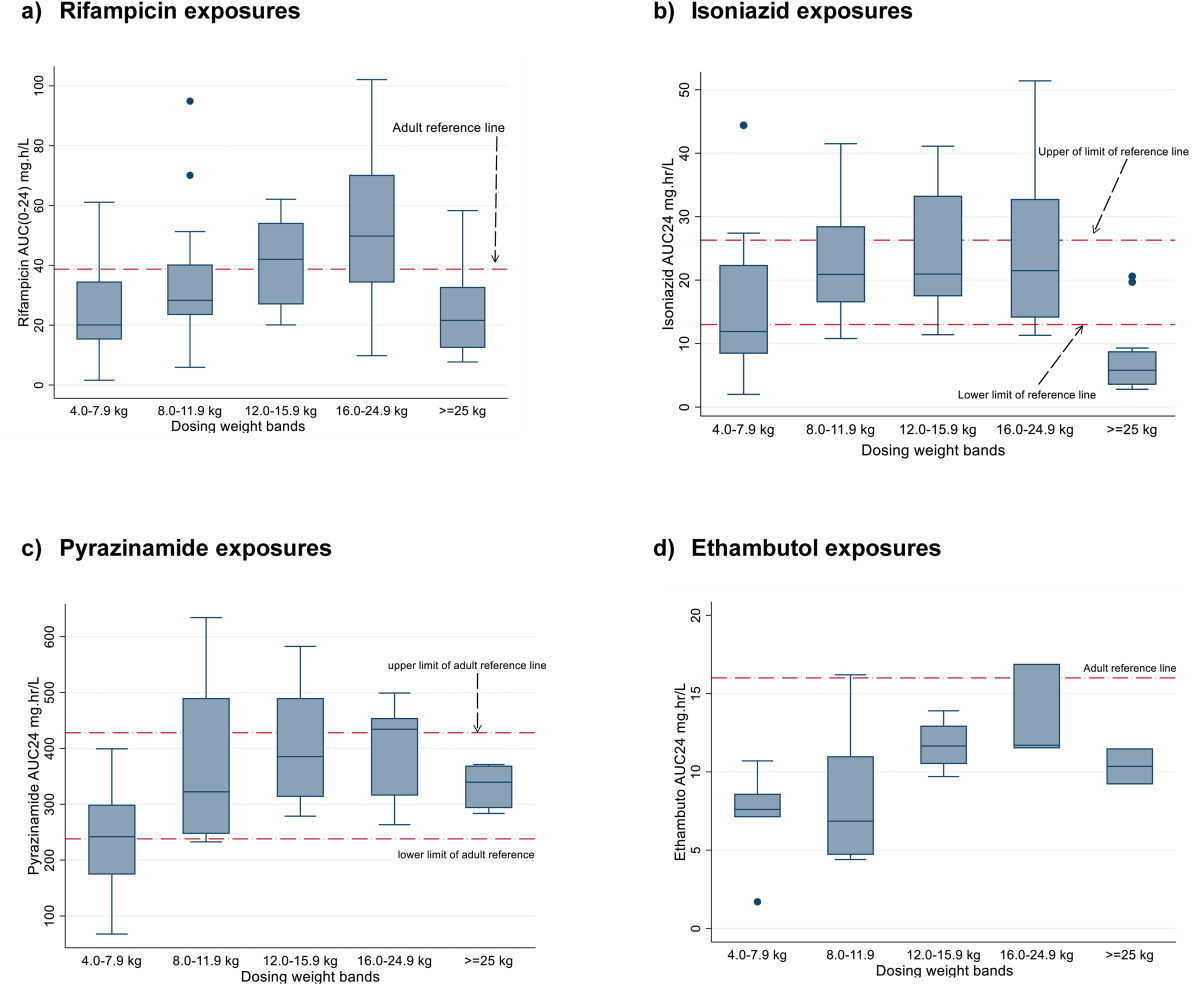
Box plots for the area under the concentration-time curve from 0 to 24 hours (AUC_24_) for rifampicin, isoniazid, pyrazinamide, and ethambutol in children treated for tuberculosis, by weight band. Horizontal reference lines represent target exposures derived from adult studies. For rifampicin, the estimated AUC_24_ (38.73 mg⋅h/L) was derived from a systematic review and meta-analysis by Stott et al [22]. For isoniazid, pyrazinamide, and ethambutol, 11.6–26.3, 233–429, and 16–28 mg/L represent the respective ranges of the medians from studies in a systematic review by Daskapan et al [23].

**Figure 2.**
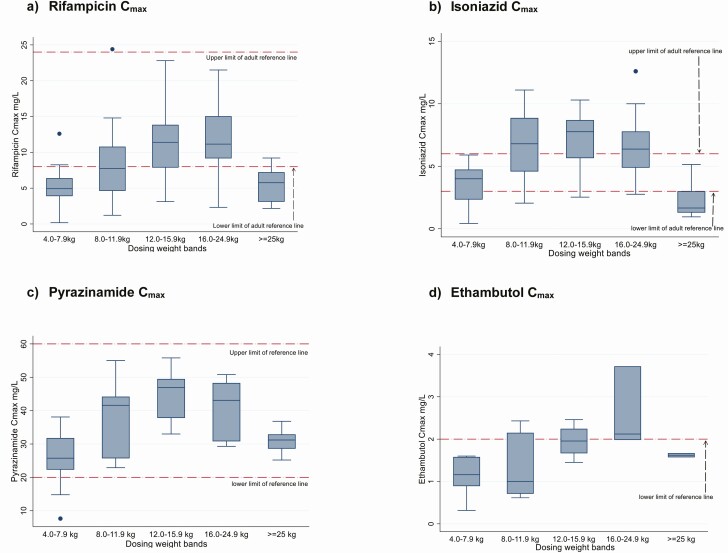
Box plots for rifampicin, isoniazid, pyrazinamide, and ethambutol peak concentration (C_max_) in children treated for tuberculosis, by weight band. Horizontal reference lines represent the target reference ranges for C_max_ recommended by Alsultan and Peloquin [16]: 3–6 mg/L, 8–24 mg/L, 20–60 mg/L, and 2–6 mg/L for isoniazid, rifampin, pyrazinamide, and ethambutol, respectively.

The median AUC_24_ (IQR) for rifampicin was 32.5 (20.1–45.1) mg⋅h/L. Most children in the 4.0–7.9, 8–11.9, and ≥25.0 kg weight bands had exposures below the adult reference (Tables 3 and 4 and Figure 1). Regression analysis of factors affecting the AUC_24_ after adjusting for weight band showed a trend to lower exposures with HIV infection (−10.6; 95% confidence interval −21.9 to .7; *P* = .07) (Table 5). The median C_max_ (IQR) was 7.6 (4.9–11.4) mg/L (Table 3 and Supplementary Table 1) with 40 of 77 children (52%) failing to attain a C_max_ of 8 mg/L, the lower limit of the reference range (Figure 2).

**Table 3.** Pharmacokinetic Parameters for Rifampicin, Isoniazid, Pyrazinamide, and Ethambutol in Children Treated for Tuberculosis

	Median Value (IQR)			
Parameter	Rifampicin (n = 77)	Isoniazid (n = 76)	Pyrazinamide (n = 45)	Ethambutol (n = 22)
AUC_24_, mg⋅h/L^a^	32.5 (20.1–45.1)	16.7 (9.2–25.9)	317 (263–399)	9.5 (7.5–11.5)
C_max_, mg/L	7.6 (4.9–11.4)	5.1 (2.8–7.7)	33.0 (25.9–43.1)	1.6 (0.9–2.0)
t½, h	1.7 (1.5–2.3)	3.2 (2.6–4.2)	6.3 (5.6–7.5)	4.4 (3.3–5.7)
t_max_, h	2 (1–2)	1 (1–2)	1 (1–2)	2 (2–4)
K_e_, h^−1^	0.40 (0.30–0.47)	0.22 (0.16–0.26)	0.11 (0.09–0.12)	0.16 (0.12–0.21)

Abbreviations: AUC_24_, area under the concentration-time curve from 0 to 24 hours; C_max_, maximum plasma concentration; IQR, interquartile range; K_e_, elimination rate constant; t_½_, elimination half-life; t_max_, time to C_max_.

^a^The following AUC_24_ reference values were used. For rifampicin, the estimate AUC_24_ (38.73 mg⋅h/L) is derived from a systematic review and meta-analysis by Stott et al [22]. For isoniazid, pyrazinamide, and ethambutol, 11.6–26.3, 233–429, and 16–28 mg⋅h/L represent the respective ranges of the medians from studies in a systematic review by Daskapan et al [23]. The target C_max_ reference ranges recommended by Alsultan and Peloquin [16] were 8–24 mg/L, 3–6 mg/L, 20–60 mg, and 2–6 mg/L for rifampicin, isoniazid, pyrazinamide, and ethambutol, respectively.

**Table 4.** Median Area Under the Concentration-Time Curve for Rifampicin, Isoniazid, Pyrazinamide, and Ethambutol, by Weight Band in Children Treated for Tuberculosis

	AUC_24_, Median (IQR), mg⋅h/L			
Weight Band, kg	Rifampicin (n = 77)	Isoniazid (n = 76)	Pyrazinamide (n = 45)	Ethambutol (n = 22)
4–7.9	20.1 (15.2–34.6) [n = 16]	11.9 (8.4–22.4) [n = 16]	242 (174–299) [n = 14]	7.6 (7.1–8.6) [n = 7]
8–11.9	28.3(23.4– 40.3) [ n = 14]	20.9 (16.5–28.5) [n = 14]	322 (247–490) [n = 11]	6.9 (4.7–11.0) [n = 6]
12–15.9	42.0 (27.0–54.2) [n = 16]	21.0 (17.5–33.3) [ n = 16]	385 (313–490) [n = 7]	11.6 (10.5–13.0) [n = 4]
16–24.9	49.8 (34.3–70.3) [n = 16]	21.5 (14.1–32.8) [n = 15]	434 (315–454) [n = 7]	11.7 (11.5–16.9) [n = 3]
≥25	21.6 (12.4–32.8) [n = 15]	5.8 (3.5–8.8) [n = 15]	339 (293–369) [n = 6]	10.4 (9.2–11.5) [n = 2]

Abbreviations: AUC_24_, area under the concentration-time curve from 0 to 24 hours; IQR, interquartile range.

**Table 5.** Area Under the Concentration-Time Curve for Rifampicin, Isoniazid, Pyrazinamide, and Ethambutol in Children Treated for Tuberculosis, by Patient Characteristics

		Rifampicin		Isoniazid		Pyrazinamide		Ethambutol	
Patient Characteristic	Children Evaluated, No.	Coefficient (95% CI)^a^	P Value	Coefficient (95% CI)^a^	P Value	Coefficient (95% CI)^a^	P Value	Coefficient (95% CI)^a^	P Value
Sex									
Male	40	Referent	.88	Referent	.67	Referent	.63	Referent	.62
Female	37	0.8 (− 9.3 to 10.9)		1.2 (−4.4 to 6.8)		20.2 (−63.0 to 103)		−1.1 (−5.7 to 3.5)	
HIV status									
Negative	47	Referent	.07	Referent	.18	Referent	.97	Referent	.83
Positive	20	−10.6 (−21.9 to .7)		−3.9 (−9.7 to 1.9)		−4.8 (226.5, 216.9)		−.9 (−9.9 to 8.1)	
WAZ									
Below −2	29	Referent	.59	Referent	.75	Referent	.61	Referent	.04
≥2	48	3.2 (−8.7 to 15.1)		−1.0 (−7.1 to 5.1)		23.9 (−71.2 to 119)		4.2 (.2–8.2)	
WHZ									
Below −2	52	Referent	.56	Referent	.87	Referent	.54	Referent	.52
≥2	8	−5.5 (−24.2 to 13.2)		1.0 (−11.0 to 13.0)		52.4 (−120 to 226)		−2.2 (−9.4 to 5.0)	

Abbreviations: CI, confidence interval; HIV, human immunodeficiency virus; WAZ, weight-for-age *z* score; WHZ, weight-for-age *z* score.

^a^Adjusted for weight band.

The isoniazid pharmacokinetic profile for 1 child was not analyzable and was excluded. The median AUC_24_ (IQR) was 16.7 (9.2–25.9) mg⋅h/L, within the adult reference range (Tables 3 and 4 and Figure 1). Children ≥25.0 kg receiving adult formulations had a median AUC_24_ of 5.8 mg⋅h/L, about half the lowest study median of the adult target range. Low AUC_24_ values were also observed in the extreme weight bands (4.0–7.9 and ≥25 kg), compared with adult references. Sex, HIV status, and anthropometric measures were not associated with AUC_24_ after adjustment for weight band (Table 5). The median C_max_ (IQR) for isoniazid was 5.1 (2.8–7.6) mg/L. The C_max_ was below the reference range for 22 of 76 children (29%), while 31 (41%) had a C_max_ >6 mg/L (Table 3, Supplementary Table 1, Figure 2 and Supplementary Figure 2).

The median AUC_24_ (IQR) for pyrazinamide was 317 (263–399) mg⋅h/L (Table 3) and within the adult reference range across the weight bands (Tables 3 and 4 and Figure 1). AUC_24_ was not significantly associated with sex, HIV status, or anthropometric measures after adjustment for weight band (Table 5). The median C_max_ (IQR) was 33.0 (25.9–43.1) mg/L, within the reference range in 40 of 45 children (89%) Table 3, Supplementary Table 1, and Figure 2). The ethambutol AUC_24_ (IQR) was 9.5 (7.5–11.5) mg⋅h/L, with the median AUC_24_ well below the adult reference range for all weight bands (Tables 3 and 4 and Figure 1). Low WAZ (below −2) was associated with lower AUC_24_ after adjustment for weight bands (Table 5). The median C_max_ (IQR) for ethambutol was 1.6 (0.91–2.0) mg/L, with values below the recommended reference range in 16 of 22 children (73%) (Table 3, Supplementary Table 1, and Figure 2). For all drugs, the exposures increased with increasing weight band, except for children ≥25 kg (Figure 1), and C_max_ and AUC_24_ were strongly correlated (rifampicin, *r* = 0.87; isoniazid *r* = 0.79; pyrazinamide *r* = 0.87; and ethambutol, r = 0.88; *P* < .01 for all) (Supplementary Figure 1).

## DISCUSSION

This is the first pharmacokinetic study to assess the WHO’s weight band–based dosing using child-friendly pediatric FDCs that are now widely available in low and middle-income countries as the preferred formulations for young children. We found that rifampicin exposures were low; in the lowest weight band (4.0–7.9 kg), values were about half of those observed in adults, and they were also low in the 8.0–11.9-kg weight band. Ethambutol exposures were low in all weight bands. Exposures of all the drugs increased with increasing weight band, except for children ≥25 kg receiving adult doses, who had very low rifampicin, isoniazid, and ethambutol AUC_24_. Only 48% and 27% of the children achieved C_max_ values above the lower limit of the recommended adult ranges for rifampicin and ethambutol, compared with 70% and 89% for isoniazid and pyrazinamide, respectively.

Our findings are consistent with those of other studies in children treated with the revised WHO doses, who did not achieve recommended concentrations of rifampicin [17–19, 31, 32] and ethambutol [17–19, 32] but had adequate concentrations for isoniazid and pyrazinamide [17–19]. In contrast to these studies, we used the child-friendly FDCs with a rifampicin-isoniazid ratio of 3:2, currently recommended by the WHO. Our results suggest that higher doses (in milligrams per kilogram) should be used in smaller children to achieve current adult drug exposure targets. Lower milligram-per-kilogram exposures in the 4.0–7.9-kg weight band could be partly because most children weighed near the upper end of the weight band. However, similar observations in a study of Malawian and South African children support our finding that drug concentrations are low in children weighing <8 kg, except in infants aged <3 months, who have immature metabolic pathways [33]. 

Young children are most vulnerable to severe forms of tuberculosis and may have worse treatment outcomes than older children [34]. Under current dosing guidelines, the smallest children have the lowest drug exposures, which might be critical in those with severe or extensive disease, including children with disseminated tuberculosis. We also showed low drug exposures in children 25–36.9 kg who receive lower milligram-per-kilogram rifampicin and isoniazid doses than children weighing <25 kg. These results support proposals to increase the first-line antituberculosis drug doses currently recommended for adults weighing <55 kg, using HRZE (150/75/400/275-mg FDC) [20].

The SHINE trial results showed that the 4-month regimen was noninferior to 6 months of treatment, with excellent treatment outcomes in children with non-severe tuberculosis across the randomization arms. In the 1204 children enrolled, unfavorable outcomes were few (7% in the intention-to-treat population including treatment failure, tuberculosis recurrence, loss to follow-up, and all-cause mortality), and only 17 grade 3 or more treatment-related adverse events were reported, of which 11 were raised liver enzyme levels and 10 led to treatment interruption or discontinuation [35]. Notably, SHINE did not include children with severe or extensive disease. Optimized dosing may further improve tuberculosis treatment outcomes in children across the whole severity spectrum of tuberculosis disease.

The reference AUC_24_ used for rifampicin should be regarded as a minimum target for the average exposure in each of the pediatric weight bands. The reference is based on the mean AUC_24_ (38.73 mg⋅h/L) derived by Stott et al [22] in a meta-analysis of pharmacokinetic studies that provides the most comprehensive assessment of exposures in adults. The corresponding mean C_max_ of 5.79 mg/L is well below the widely applied recommended range for C_max_ (8–20 mg/L) with standard treatment [16].

There is growing interest in the use of high-dose rifampicin. Preliminary studies in adults with drug-susceptible tuberculosis suggest that rifampicin doses as high as 35 mg/kg are tolerated well, improve antituberculosis activity, and could potentially lead to treatment shortening [36, 37]. The establishment pediatric rifampicin doses that would match the exposures observed in adults dosed at 35 mg/kg is currently under evaluation in the OptiRif study [38]. With optimized doses, it is possible that treatment shortening, shown to be effective, feasible, and safe in children with non-severe tuberculosis in SHINE, could also be achieved in children with other forms of tuberculosis, including those with severe or extensive tuberculosis disease.

Except in the lowest and highest weight bands, adequate isoniazid exposures were in keeping with findings of studies evaluating the revised doses [17–19]. One Indian study reported higher-than-normal AUC and C_max_ in children dosed at 10 mg/kg. In SHINE, the pharmacokinetics of the new FDCs in Indian children will be analyzed, once the validation process of the assays used in Indian sites is completed. Further pharmacogenomic studies are planned to evaluate the impact of slow acetylator status. For pyrazinamide, the finding of levels comparable to those in adults is reassuring and consistent with findings of other studies [17, 18, 31].

Ethambutol was used in only a third of the children in this study, based on local guidelines. In keeping with other studies in children [17–19, 31], we found low AUC_24_ and C_max_ values across all weight bands. With such low systemic exposures, it is uncertain whether ethambutol prevents the development of resistance to other drugs in circumstances of primary isoniazid resistance. The fact that, optic neuritis is rarely observed in children may be partly due to low ethambutol exposures [9]. No clinically significant ocular toxicity was reported in the SHINE trial which used color vision testing in children aged ≥3 years [39]. The risk of ocular toxicity is dose dependent, and if the higher doses were used, that risk would need to be reevaluated [9].

The considerable variation in drug exposure by weight band in our study was in part due to the assumption that uniform milligram-per-kilogram doses are required regardless of body size. Allometric scaling is increasingly used to estimate the higher milligram-per-kilogram requirements of smaller children, to avoid systematic underdosing [40]. There is a wide range of milligram-per-kilogram doses within a weight band, most notably in the lower bands, resulting in additional variability [33]. In addition, immaturity of phase I/II drug metabolizing enzymes leads to higher drug exposures in young infants, particularly those <3 months old [24, 33, 41]. 

Owing to high correlation of milligram-per-kilogram doses with weight band, we did not evaluate the separate impact of these doses on drug exposure. The average exposure in each weight band is therefore dependent on the distribution of the participants’ body weights, which may or may not accurately represent the weight distribution of children treated for tuberculosis in other settings. We did not confirm an association between HIV coinfection status and lower antituberculosis drug concentrations [21]. Poor nutritional status has been linked to lower drug concentrations [18, 32, 42]. Except for the association between WAZ score and ethambutol AUC_24_, we did not find significant associations between the drug exposures and anthropometric measurements or study site. Population pharmacokinetic modeling is planned, and genetic polymorphisms have not been assessed in this study.

A growing literature on optimal tuberculosis drug exposures based on pharmacokinetic-pharmacodynamic studies suggest alternative targets in many instances [12, 16, 20, 43]. However, until these are validated as optimal as part of combination treatment, target ranges based on the exposures encountered in adults receiving standard doses are an accepted approach. Our study was not designed to evaluate whether disease severity affects pharmacokinetics. A 2020 study found significantly lower antituberculosis drug exposures in adults with severe tuberculosis-HIV disease, while another found no important pharmacokinetic differences between hospitalized patients and their ambulatory counterparts [44, 45].

In the context of standardized dosing of the antituberculosis drugs for children, drug exposures should match those considered optimal in adults. The revised WHO 2010 recommendations result in improved antituberculosis drug exposures in children, but target exposures are still not achieved across all weight bands. Of particular concern are the relatively low rifampicin exposures in the extreme range weight bands. The role of ethambutol in first-line antituberculosis treatment in children should be investigated as the contribution of ethambutol at the very low exposures found in children with currently used doses is uncertain.

## Supplementary Data

Supplementary materials are available at *Clinical Infectious Diseases* online. Consisting of data provided by the authors to benefit the reader, the posted materials are not copyedited and are the sole responsibility of the authors, so questions or comments should be addressed to the corresponding author.

ciab725_suppl_Supplementary_Figure_Legends

ciab725_suppl_Supplementary_Figure_S1

ciab725_suppl_Supplementary_Figure_S2

ciab725_suppl_Supplementary_Table_S1

## Notes

***Author contributions.*** C. C. and H. M. prepared the manuscript and coordinated the writing of it. C. C., M. C., and H. M. analyzed and interpreted the pharmacokinetic analysis. L. W. supervised the laboratory analysis of the drug assays. A. T., R. A., D. G., and H. M. designed the study and made key revisions to the draft manuscripts. A. C. H., K. M. Z., M. v. d. Z., M. K., M. P., and V. M. implemented the pharmacokinetic study at the SHINE sites in Zambia and South Africa. All authors reviewed the manuscript for intellectual content and approved the final version of the report.

***Acknowledgments.*** The SHINE team thanks all the staff in all the participating sites and the children and families who took part in the trial.

***Financial support.*** This work was supported by the Joint Global Health Trials Scheme of the Department for International Development, United Kingdom, the Wellcome Trust and the UK Medical Research Council (grant MR/L004445/1), the TB Alliance, the UK Medical Research Council (core support; grants MC_UU_12023/26 and MC_UU_12023/27), the Wellcome Trust (grant 206379/Z/17/Z to H. M.), the Adult Clinical Trial Group (support to Cape Town Clinical PK Laboratory), the National Institute of Allergy and Infectious Diseases, National Institutes of Health (grants UM1 AI068634, UM1 AI068636, and UM1 AI106701 to Cape Town Clinical PK Laboratory and grant U01 AI068632 through the Infant Maternal Pediatric Adolescent AIDS Clinical Trials Group), the Eunice Kennedy Shriver National Institute of Child Health and Human Development, and the National Institute of Mental Health (grant AI068632).

***Potential conflicts of interest.*** C.C and A. C. H. report receiving institutional funding to participate as SHINE trial sites from UK Medical Research Council (MRC)-Clinical Trials at University College, London, through a prime grant award from UK MRC, Wellcome Trust, and Department for International Development (grant number MR/L004445/1). A. T., L. C., K. L., and D. G. report COVID 19 Grant Extension Allocation award 181573 from UK Research Innovation. V. M. reports grants or contracts with the National Institutes of Health, outside the submitted work. All other authors report no potential conflicts. All authors have submitted the ICMJE Form for Disclosure of Potential Conflicts of Interest. Conflicts that the editors consider relevant to the content of the manuscript have been disclosed.
