# Supplementary material for: Pharmacokinetics of First-Line Drugs in Children With Tuberculosis, Using World Health Organization–Recommended Weight Band Doses and Formulations
Source: Clin Infect Dis. 2021 Aug 22;74(10):1767–75. doi: 10.1093/cid/ciab725 (PMC9155615; doi:10.1093/cid/ciab725)
Supplement: ciab725_suppl_Supplementary_Figure_Legends [file ciab725_suppl_supplementary_figure_legends.docx]

**Supplementary figure legend:**

**Figure S1: Correlation between AUC24 and Cmax for rifampicin, isoniazid, pyrazinamide and ethambutol in children treated for tuberculosis**

**Figure S2: Distribution of isoniazid half-life in children treated for tuberculosis**
