# Supplementary material for: Pharmacokinetics of First-Line Drugs in Children With Tuberculosis, Using World Health Organization–Recommended Weight Band Doses and Formulations
Source: Clin Infect Dis. 2021 Aug 22;74(10):1767–75. doi: 10.1093/cid/ciab725 (PMC9155615; doi:10.1093/cid/ciab725)
Supplement: ciab725_suppl_Supplementary_Table_S1 [file ciab725_suppl_supplementary_table_s1.docx]

**Table S1: Median (IQR) area peak concentrations (C_max_) for rifampicin, isoniazid, pyrazinamide and ethambutol summarized by weight band in children treated for tuberculosis**

|  | **Rifampicin (N=77)** | | **Isoniazid (N=76)** | | **Pyrazinamide (N=45)** | | **Ethambutol (N=22)** | |
| --- | --- | --- | --- | --- | --- | --- | --- | --- |
| **Weight-band, (kg)** | n | C_max_, (mg/L) | n | C_max_, (mg/L) | n | C_max_, (mg/L) | n | C_max_, (mg/L) |
| **4-7.9** | 16 | 4.9 (3.9 – 6.4) | 16 | 4.0 (2.3 – 4.7) | 14 | 25.8 (22.3 – 31.8) | 7 | 1.2 (0.9 – 1.6) |
| **8-11.9** | 14 | 7.7 (4.6- 10.8) | 14 | 6.8 (4.6 – 8.9) | 11 | 41.6 (25.7 – 44.2) | 6 | 1.0 (0.7 – 2.2) |
| **12-15.9** | 16 | 11.4 (7.9 – 13.9) | 16 | 7.8 (5.7 – 8.7) | 7 | 46.9 (37.8 – 49.5) | 4 | 2.0 (1.7 – 2.4) |
| **16-24.9** | 16 | 11.2 (9.2 – 15.1) | 15 | 6.4 (4.9 – 7.8) | 7 | 43.1 (30.8 – 48.3) | 3 | 2.1 (2.0 – 3.7) |
| **≥25** | 15 | 5.8 (3.1 – 7.2) | 15 | 1.7 (1.3 – 3.0) | 6 | 31.2 (28.6 – 32.9) | 2 | 1.6 (1.5 – 1.7) |

IQR=Interquartile range, C_max_=peak plasma concentration. Target reference ranges for C_max_ recommended by Alsultan *et al* [16]: rifampicin 8-24mg/L, isoniazid 3-6mg/L, pyrazinamide 20-60mg and ethambutol 2-6mg/L.
